# Identification and in silico analysis of a spectrum of *SLC4A11* variations in Indian familial and sporadic cases of congenital hereditary endothelial dystrophy

**DOI:** 10.1186/s13023-022-02521-4

**Published:** 2022-09-17

**Authors:** Mohd Salman, Anshuman Verma, Sunita Chaurasia, Deeksha Prasad, Chitra Kannabiran, Vivek Singh, Muralidhar Ramappa

**Affiliations:** 1 Prof. Brien Holden Eye Research Center, Champalimaud Translational Centre for Eye Research, L V Prasad Eye Institute, Hyderabad, India; 2 Manipal Academy of Higher Education, Manipal, Karnataka India; 3 MNR Foundation for Research and Innovations, MNR Medical College, MNR Nagar, Sangareddy, Telangana India; 4 Centre for Rare Eye Diseases and Ocular Genetics, L V Prasad Eye Institute, Hyderabad, India; 5 Jasti V Ramanamma Children’s Eye Care Center, L V Prasad Eye Institute, Hyderabad, India; 6 The Cornea and Anterior Segment, L V Prasad Eye Institute, KAR Campus, Banjara Hills, Hyderabad, India

**Keywords:** CHED, *SLC4A11*, Corneal dystrophy, Variations

## Abstract

**Background:**

Congenital hereditary endothelial dystrophy (CHED) is a rare form of corneal dystrophy caused by *SLC4A11* gene variations. This study aims to find the genetic alterations in *SLC4A11,* in two Indian familial CHED cases with affected members n = 3 and n = 2 respectively and five sporadic CHED cases using direct sequencing, followed by in silico analysis and characterization of the identified variants.

**Results:**

All three affected members of the first CHED family were identified with a novel homozygous c.1514C > G (p.Ser489Trp) variation while second family showed presence of a compound heterozygous variation c.529A > C (p.Arg161Arg) + c.2461insT (p.Val805fs). Among five sporadic cases, two showed novel changes, homozygous c.1487G > T (p.Ser480Ile) and c.620-2A > G, while the other one had previously reported homozygous c.2653C > T (p.Arg869Cys) variation. The remaining two cases did not reveal the presence of SLC4A11-related pathogenic variations. The identified variations were excluded from the Indian control (n = 80). In silico analysis using homology-based protein modeling and pathogenicity prediction tools, which revealed these alterations as pathogenic, changing their protein stability, local flexibility, residue contact clashes, and the hydrogen bond interactions.

**Conclusions:**

This study contributed to the CHED mutational spectrum, adding four novel variations and confirming a previously reported one. It demonstrates different type of variations in CHED cases, including coding, non-coding, homozygous, synonymous, and compound heterozygous variations. The identified variations revealed different degrees of pathogenic effects in silico. Moreover, two sporadic cases could not be identified with pathogenic variation emphasizing the involvement of other genes or genetic mechanisms.

**Supplementary Information:**

The online version contains supplementary material available at 10.1186/s13023-022-02521-4.

## Background

Congenital hereditary endothelial dystrophy (CHED) [MIM: 217700] is a rare corneal dystrophy and holds a significant indication of corneal transplantation in India and middle east countries [1]. The disease causes dysfunction in the corneal endothelium, presenile senescence of corneal endothelial cells, a thickened Descemet membrane, and progressive corneal clouding in the early stages of life. Patients may present with varying degrees of amblyopia, nystagmus, and glaucomatous features in the advanced stages of the disease [2]. Genetic causes of CHED have been identified in British [3] and Irish [4] familial cases using linkage analysis, describing CHED1 (autosomal dominant) and CHED2 (autosomal recessive) with a common genetic locus. Later studies distinguished them as two separate loci [5]. A study by Vithana et al. in a consanguineous family from Myanmar refined the CHED2 locus and confirmed the underlying genetic factor, the solute carrier family 4 member 11 (*SLC4A11*) as the prime candidate gene [6]. Subsequent studies confirmed these findings, and many *SLC4A11* mutations were reported (Additional file 1: Table S1) establishing *SLC4A11* as the causative gene for CHED2. *SLC4A11* knockout (K/O) mice also corroborate with the CHED phenotype, confirming its role in disease pathogenesis [7]. In the current international classification of corneal dystrophy (IC3D), CHED2 is an autosomal recessive form of corneal endothelial dystrophy or CHED. CHED1 is the posterior polymorphous corneal dystrophy (PPCD) or posterior polymorphous dystrophy part of an autosomal dominant form of corneal endothelial dystrophy [8]. The *SLC4A11* gene, located on chromosome 20p13, encodes for a transmembrane sodium bicarbonate transporter-like protein-11. On the corneal endothelial surface, it acts as a “pump” to transport ions across the endothelium to the corneal stroma. It helps in water movement from the stroma to the aqueous humour, maintaining the dehydrated state of the corneal stroma [9]. Apart from CHED, a mutation in *SLC4A11* also causes a form of autosomal dominant Fuch’s corneal endothelial dystrophy (FECD) and Harboyan syndrome or corneal dystrophy-perceptive deafness [10]. The clinical CHED phenotype overlaps with primary congenital glaucoma and PPCD, whose genetic origins are different. Screening for *SLC4A11* variations can help in the differential and confirmative diagnosis of CHED to manage the available corneal transplant options, including Descemet’s membrane endothelial keratoplasty (DMEK) and Descemet’s stripping automated endothelial keratoplasty (DSAEK) [11]. Some *SLC4A11* single nucleotide polymorphisms (SNPs) are linked with bisphenol A induced ovarian carcinoma [12]. Specific non-steroidal anti-inflammatory drugs are being investigated for correcting *SLC4A11*-specific mutant effects [13]. The *SLC4A11* mutational spectrum can help to develop medicinal approaches and regenerative medicine, such as Gene Therapy (GT) or Clustered Regularly Interspaced Short Palindromic Repeats (CRISPR) based gene editing, [14–16]. Also showed in our recent publication on corneal [17]. Screening for familial CHED cases also helps to measure the risk and manage the disease. This study analysed two familial cases of CHED with multiple affected members and five sporadic cases, screening all exons with flanking intronic regions of the *SLC4A11* gene using the direct sequencing method. We also characterized the identified variations in silico using homology modelling and pathogenicity prediction tools and looked at the disease phenotype.

## Methods

### Ethics statement

This study was approved by the institutional review board of the LV Prasad Eye Institute (Ethics Ref. No. LEC-BHR-01–20-381) and followed the tenets of the declaration of Helsinki.

### Subjects and clinical examination

Patients were recruited at the LV Prasad Eye Institute, India, between 2007 and 2021. Every participant, or the guardian of a minor, signed the informed consent. The demographic and clinical details of the participants are shown in Table 1. The mean age of the affected individuals at presentation was 9.6 y with a range of 7–15 y. The majority were females (n = 6) and males (n = 4). The condition was diagnosed based on ground glass corneal clouding in the presence of normal anterior segment architecture. All the patients had a detailed ophthalmic examination, including a slit lamp, optical coherence tomography, and specular microscopic imaging. The corneal opacity was classified as mild with visible iris, moderate obscuring iris details, and severe obscuring the intraocular structure details.

**Table 1:** Details of the CHED cases recruited to this study

CHED Case ID familial (F) and sporadic (N)	Onset of disease	Age	Sex	Visual acuity		Corneal thickness (µm) OD/OS	Corneal opacity	Surgical interventions
				OD	OS			
F1a	Early adolescence	14	Female	CF 1 m	CF/CF	989/974	Severe	DSAEK + MMC
F1b	Middle childhood	10	Female	20/400	20/600	961/742	Moderate	DSAEK
F1c	Middle childhood	7	Male	20/600	20/600	986/940	Severe	DSAEK
F2a	Middle childhood	14	Female	20/800	20/800	1310/1280	Severe	DSAEK + MMC
F2b	Early adolescence	9	Male	20/160	20/200	1200/1210	Mild	DSAEK
N1	Early adolescence	13	Female	20/160	CF/CF	1040/1110	Moderate/Severe	DSAEK
N2	Early adolescence	7	Female	20/800	20/800	967/995	Severe	DSAEK
N3	Middle childhood	12	Male	20/160	20/160	1080/1100	Moderate	DSAEK
N4	Early adolescence	15	Female	CF/CF	CF/CF	1200/1220	Severe	DSAEK
N5	Middle childhood	8	Male	20/400	20/500	781/776	Moderate	DSAEK

*OD* Oculus Dexter (Right eye); *OS* Oculus sinister (Left eye); *CF* Counting fingers; *DSAEK* Descemet’s stripping automated endothelial keratoplasty; *MMC* Mytomycin C

**Fig. 1:**
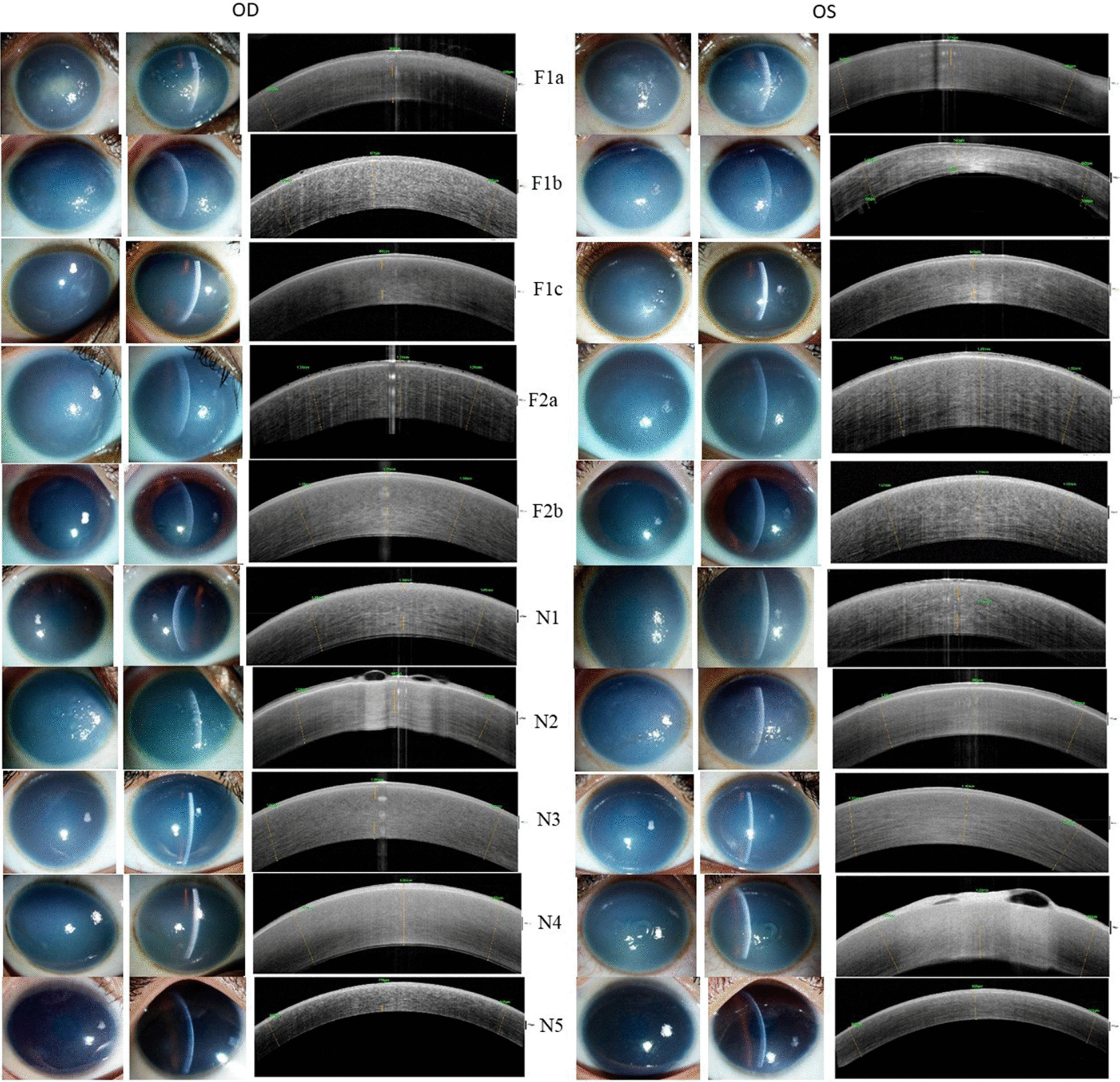
Clinical slit lamp and anterior segment-optical coherence tomography (AS-OCT) images of the eyes of the recruited participants. Left to right: Slit lamp and AS-OCT images of the familial (F1 and F2) and sporadic (N1-N5) CHED cases. A cloudy and hazy cornea is observed using the slit lamp. The OCT images indicating edematous haze in the individuals. OD: Right eye; OS: Left Eye

**Fig. 2:**
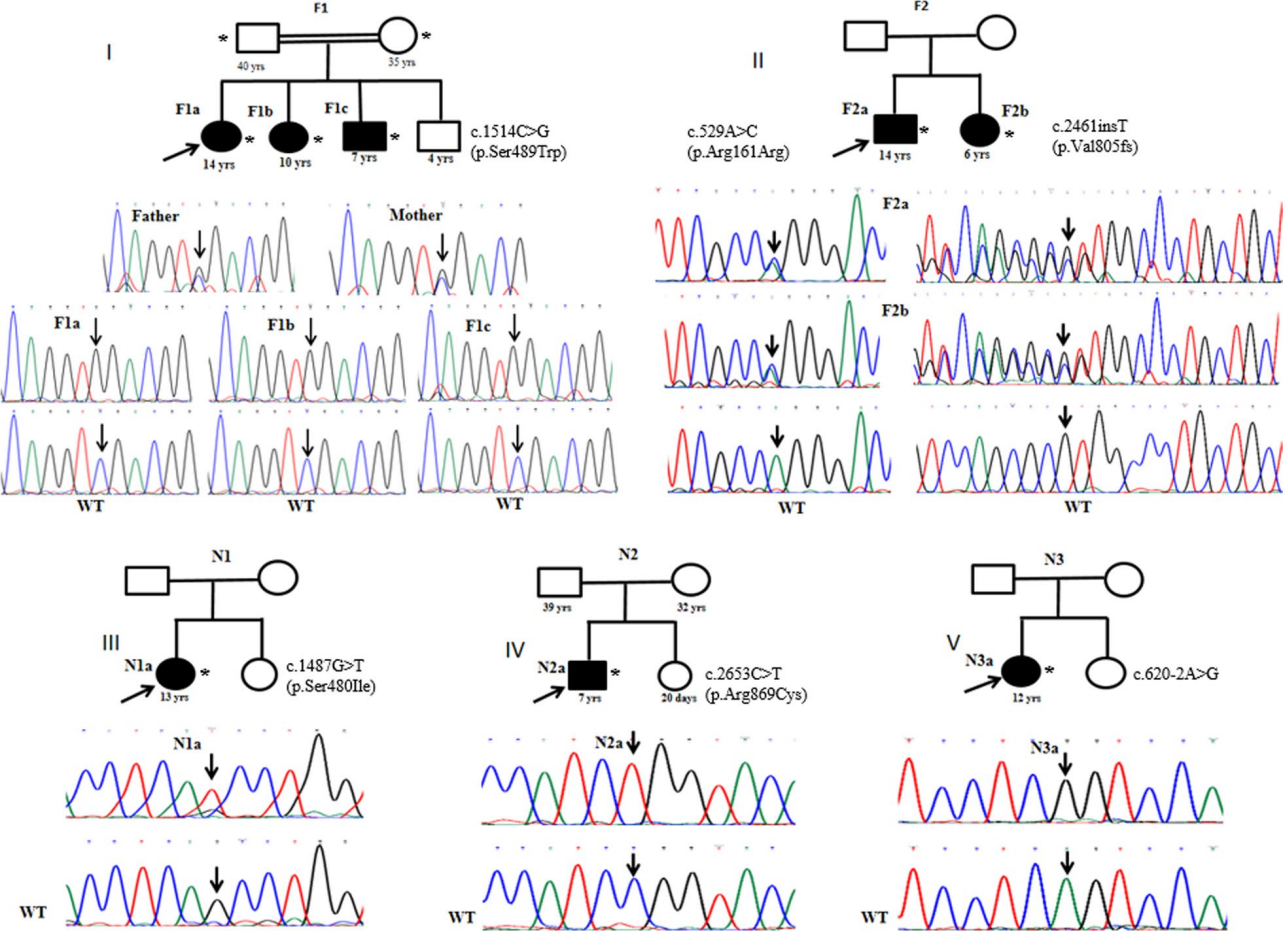
Pedigree and chromatograms of familial and sporadic CHED cases identified with *SLC4A11* variations. Panels I-V show the representative pedigrees and their sequenced chromatograms for all the CHED cases. The members underwent sequencing analysis are marked with asterisked*.The affected members sequence chromatogram were compared with the wild-type (WT) chromatogram (bottom). I and II represent familial (F1 and F2) cases identified with homozygous c.1514C>G (p.Ser489Trp) and compound heterozygous c.529A>C (p.Arg161Arg) + c.2461insT (p.Val805fs) variations, respectively. II, III, and IV represent sporadic cases (N1, N2, and N3) identified with homozygous c.1487G>T (p.Ser480Ile), c.2653C>T (p.Arg869Cys), and c.620-2A>G variations, respectively

The increase in corneal thickness, caused by edema, was measured using Fourier domain optical coherence tomography (FD-OCT) RTVue-100 (Optovue, Fremont, California, USA) (Fig. 1). Pedigrees were constructed for each affected individuals based on the information provided by the guardians (Fig. 2). Eighty healthy individuals from the Indian population, (47.5% males, 52.5% females) (mean age- 31.75y) without any clinical history of ocular dystrophy were recruited as control.

### Genetic analysis

For each participant**,** 2–3 mL of blood was drawn from a radial vein. The parents were also genetically screened along with the affected individuals of the family. The genomic DNA was extracted from blood samples using a DNA extraction kit (JetFlex^™^ Genomic DNA Purification Kit, A30700, Invitrogen USA). Nineteen coding exons and flanking intronic regions of the corneal specific gene transcript *SLC4A11*-201 (NCBI reference sequence: NM_032034.3) (Transcript ID: ENST00000380056.7) were targeted using 13 sets of published [18] and newly designed primers. PCR amplification of each amplicon was performed using EmeraldAmp MAX HS PCR Master Mix (RR330, Takara, Japan) as described in Additional file 1: Table S2. The diluted (1/10 to 1/15) amplified PCR product was subjected to Sanger sequencing using a BigDye Terminator ready reaction mix on an ABI-3130XL sequence analyser (Applied Biosystems, USA). The sequence chromatograms were analysed using CHROMAS/FINCH TV [19], and the manual DNA sequence annotation was conducted using ApE [20]. The location and position of the identified variations were mapped against the ENSEMBL genome browser (https://www.ensembl.org/).

### In silico analysis

**Fig. 3:**
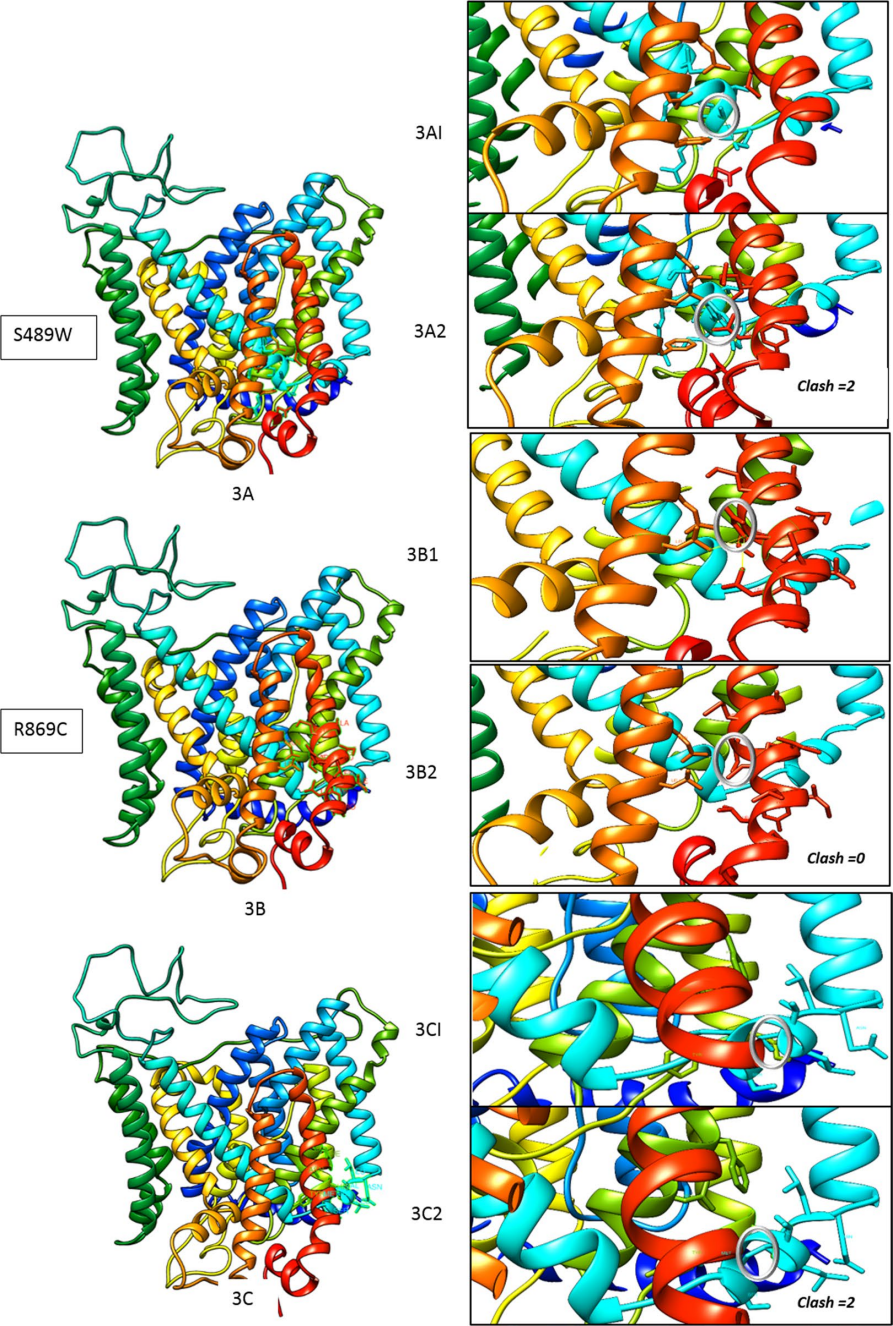
Homology-based protein modelling of *SLC4A11* protein and structural analysis. 3A, 3B, 3C shows modelled WT SLC4A11 structures in which zone around site of alteration is analysed. 3A1, 3B1,3C1 shows focused zone around WD residue and 3A2,3B2,3C2 shows corresponding focused zone around altered residue for S489W, R869C and S480I variations respectively (encircled). Number of amino acid residue clashes in surrounding zone was found two in S489W, zero in R869C and two in S480I

**Fig. 4:**
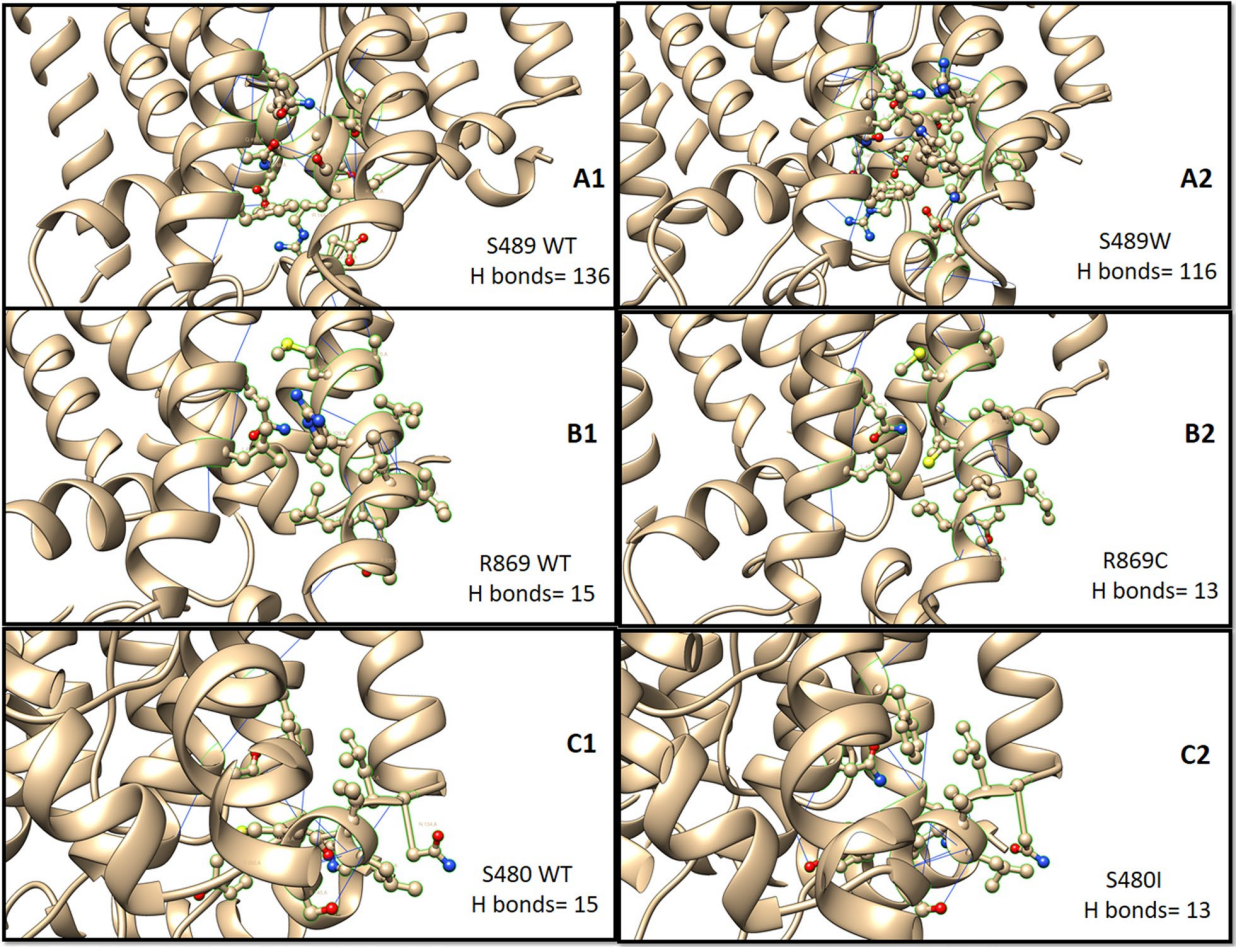
*SLC4A11* mutational effects on the number of hydrogen bond interactions around the 5 Å region of the mutation. **A1**, **B1**, and **C1** shows the number of H bonds of WT *SLC4A11* at S489, R869, and S480 positions, respectively. **A2**, **B2**, and **C2** shows the corresponding number of H bonds after it mutates to W489, C869, and I480, respectively

The pathogenicity score of the identified variations was analysed using the Predict SNP server (https://loschmidt.chemi.muni.cz/predictsnp) [21] with several tools, including SIFT [22] and PolyPhen [23]. The mutated residue conservation was analysed using the ConSurf server (https://consurf.tau.ac.il) [24] (Table 3). As the crystal protein structure of *SLC4A11* is not yet solved, a homology-based modelling approach was executed for structural analysis. Using Modeller-10.1, a basic wild type (WT) three-dimensional homology model of the human *SLC4A11* trans-membrane domain was created (Fig. 3). It was constructed using the crystal structure template of the anion exchanger domain of the human erythrocyte band-3 domain at 3.5 Å resolution (PDB 4YZF), with ~ 30% sequence identity. The structure was validated using SAVES-v6. The wild-type (WT) modelled structure was used as the template to build the mutated protein structure. The structure was visualised using CHIMERA [25]. The altered residues were analysed for any contact or clash with amino acid in the surrounding region caused due to variation (Fig. 3A1–C2). The number of hydrogen bond interactions around the 5 Å region of the variation was compared between the WT and mutated protein structures (Fig. 4). The impact on protein stability was predicted using DUET (http://marid.bioc.cam.ac.uk/sdm2/prediction, DUET (unimelb.edu.au) [26]. Protein conformation and flexibility was performed using DynaMut (http://biosig.unimelb.edu.au/dynamut/prediction) [27]. For identified intronic changes, the potential pathogenic effect for splice-site prediction was determined by the varSEAK online tool (https://varseak.bio) [28].

## Results

### Clinical and sequencing analysis

The variations identified in the two CHED familial and three sporadic cases are listed in Table 2.

**Table 2:** List of identified variations in familial and sporadic CHED cases

CHED Case familial (F) and sporadic (N)	*SLC4A11* variations identified (cDNA position)	variations identified (Protein position)	Exon/intron location	Chromosomal location	Zygosity of each individual	ACMG variants classification*	Literature status
#Family-1 (F1) (No. of affected = 3)	c.1514C > G	p.Ser489Trp	Ex.12	20:3,230,258	F1a-Hom F1b-Hom F1c-Hom Father-Het Mother-Het	Pathogenic	Not Reported
Family-2 (F2) (No. of affected = 2)	c.529A > C + c.2461insT	p.Arg161Arg + p.Val805fs	Ex.4 + Ex.17	20:3,234,173 + 20:3,228,535	F2a-Com.Het F2b-Com.Het	Pathogenic	pArg161Arg –Reported [29, 30] p.Val805fs Not Reported
Sporadic N1	c.1487G > T	p.Ser480Ile	Ex.11	20:3,230,535	N1-Hom	Uncertain significance	Not reported
N2	c.2653C > T	p.Arg869Cys	Ex.18	20:3,228,260	N3-Hom	Likely pathogenic	Reported [6, 31]
N3	c.620-2A > G	Splice variant	Int.4	20:3,233,991	N2-Hom	Pathogenic	Not Reported

Variation nomenclature was given using Transcript: ENST00000380056.7 *SLC4A11*-201

Reference: *ACMG guidelines- Richards et.al-2015 [32], ‘#’ = consanguineous marriage

#### CHED familial case 1

Three siblings presented with a CHED clinical phenotype in this consanguineous family (F1). They showed varying degrees of corneal clouding. All three cases underwent DSAEK surgery to manage their corneal dystrophy. The parents and the fourth sibling were unaffected. The *SLC4A11* sequencing identified a homozygous C to G DNA-nucleotide substitution at the 1514 position in the cDNA in all three affected cases. This caused, codon change from TCG to TGG (c.1514TCG > TGG), changing the amino acid from serine to tryptophan at the 489^th^ amino acid position (p.Ser489Trp). The parental analysis found both the parents as heterozygous for this variant, thus acting as carriers (Fig. 2I). This change was not observed in the 80 healthy Indian controls.

#### CHED familial case 2

Two siblings were affected in this non-consanguineous family (F2). The younger girl presented with significant corneal clouding, while the older boy showed a milder phenotype. The genetic analysis identified compound heterozygous changes in both siblings, comprising the substitution of A > C at the 529th position of the cDNA and insertion of a T at the 2653rd position of the cDNA (Fig. 2II). A > C at the 529th position is a synonymous change (p.Arg161Arg). Insertion of a T at the 2653rd position likely creates a frameshift at Valine in the 805th position and generates a premature stop codon at the 877th position, 15 amino acids before the natural termination codon. This change was not observed in the 80 healthy Indian controls.

#### CHED sporadic cases

Five cases (N1 to N5) with no familial history of CHED were sequenced to identify *SLC4A11* variations. Case-N1 presented with congenital bilateral decreased vision. It was identified with the c.1487 G > T variation, which was previously unreported and was not observed in the 80 Indian controls (Fig. 2III). Case-N2 showed congenital white opacity along with glaucoma. It was identified as the substitution variation, c.2653 C > T (p.Arg869Cys), reported in Indian CHED patients in two previous studies [6, 18] (Fig. 2IV). Case-N3 presented with a progressive decrease in vision along with glaucoma. It was identified as the homozygous substitution of A to G. Two bases upstream of the 620 cDNA position in the flanking intronic region at the beginning of exon 5 (Fig. 2V). This change was previously unreported and excluded in the 80 Indian controls. CHED cases N4 and N5 did not show the presence of any *SLC4A11* pathogenic changes.

### Mutational characterization—in silico analysis

#### c.1514TCG > TGG (p.Ser489Trp)

This novel variation found in family-F1 had high penetrance and persistence of occurrence as it affected three children in successive births. The wild-type Ser489 region was conserved in 147 of the 150 species protein sequences accessed. The SIFT and PolyPhen composite scores, derived from Predicts SNP, showed that the mutation has deleterious effects. The structural analysis of the mutated Trp489 residue showed two clashes in surrounding region (Fig. 3A1 and A2)). The DUET protein stability score of the mutated protein showed a destabilizing effect, and the DynaMut score indicated restricted local flexibility (Table 3). The number of hydrogen bond interactions around the 5 Å region of the mutated residues decreased from 136 to 116 (Fig. 4 A1-A2).

**Table 3:** In silico analysis of the identified non-synonymous variants in *SLC4A11*

Sr. No	Variants Identified	Residue Conservation Score (ConSurf)	Pathogenicity Score (Predict SNP pathogenicity %) [SIFT, PolyPhen2 score]								Homology modelling based analysis		
			Predict SNP	Ph.D SNP	PolyPhen2			SIFT	SNAP		H-bond interactions (5A region around the mutation site)	Protein Stability (DUET)	Protein local flexibility (DynaMut)
1	c.1514C > G (p.Ser489Trp)	Highly conserved across 147 species SCORE = 9	72%	82%		81% [1]	53% [0.002]			81%	Decreased from 136 to 116	(ΔΔG): -0.516 kcal/mol (Destabilizing)	ΔΔS_Vib_ ENCoM: -1.717 kcal.mol^−1^.K^−1^ (Decrease of molecule flexibility)
2	c.1487G > T (p.Ser480Ile)	Highly conserved across 132 species	87%	88%		81% [1]	79% [0.003]			81%	Decreased from 15 to 13	ΔΔG: -0.17 (Destabilizing)	ΔΔS_Vib_ ENCoM: -0.557 kcal.mol^−1^.K^−1^ (Decrease of molecule flexibility)
3	c.2653C > T (p.Arg869Cys)	Highly conserved across 150 species SCORE = 8	87%	82%		81% [1]	79% [0.000]			85%	Decreased from 15 to 13	ΔΔG -1.012 kcal/mol (Destabilizing)	ΔΔS_Vib_ ENCoM: 0.188 kcal.mol^−1^.K^−1^ (Increase of molecule flexibility)

SIFT score – 0–0.05: deleterious; 0.05–1: tolerated

PolyPhen2 score- 0–0.05: benign; 0.15–0.85: possibly damaging; 0.85–1: damaging

#### c.2653C > T (p.Arg869Cys)

This variation was identified in the sporadic case-N2. It was previously reported to affect the interaction of the transmembrane helix with the cytoplasmic domain of the protein [18]. The wild-type Arg869 region is conserved across all 150 species accessed. The SIFT and PolyPhen scores showed deleterious effects. The mutated Cys-869 residue showed no clashes in surrounding region (Fig. 3B1and B2). The DUET protein stability score showed the destabilizing effect of the mutation. The DynaMut score indicated increased local flexibility (Table 3). The number of hydrogen bond interactions around the 5 Å region of the mutated residue decreased from 15 to 13 (Fig. 4B1–B2).

#### c.1487G > T (p.Ser480Ile)

This novel variation was found in case-N1 in a well-conserved Ser480 position in 132 of the 150 species accessed. The Predict SNP score was deleterious. The mutated Ile 480 residue showed two clashes in surrounding region (Fig. 3C1 and C2). The mutated protein showed decreased stability and local flexibility (Table 3). The number of hydrogen bond interactions around the 5 Å region of the mutated residue decreased from 15 to 13 (Fig. 4C1–C2).

#### c.529A > C + c.2461insT (p.Arg161Arg + p.Val805fs)

Two siblings in the familial case-F2 shared this compound heterozygous change. Arg161Arg synonymous variation was previously described in both FECD cases and a few controls. It is listed as rs3827075 in the SNP database [29]. However, it was not observed in the 80 Indian controls we analysed. In this case, one heterozygous change is a synonymous change, and the other is a novel change. The prediction suggests that a combination of both changes leads to the disease phenotype.

#### c.620-2A > G

It was identified as a novel splice site variant. Two bases upstream of the exon 5 in sporadic CHED case-N3. The varSEAK online tool predicted a loss of function for the authentic splice site at this position (Table 3).

## Discussions

Ever since the *SLC4A11* gene was established as a causative gene for CHED-related phenotype, many pathogenic changes have been identified for its cause. Most of these variations (~ 75%) are single base homozygous changes in coding regions. Changes like compound heterozygous and splice site variants represent ~ 5% of the reported variations (Additional file 1 Table S1). In this study, we have identified a spectrum of changes, including substitution, compound heterozygous changes, and splice site variants in a small set of CHED cases. Among them, 80% were novel, signifying a wide range of genetic variations involved in the CHED pathogenesis. The novel variation p.Ser489Trp, identified in F1, showed different grades of severity of the disease among the three affected members. Previously, Ser489Leu alterations have been reported at the same amino acid position in CHED cases from Pakistan. An in vitro study on the Ser489Leu variant showed diffuse distribution with increased intracellular localization of the bicarbonate transporter-related protein-1 (BTR1) compared to WT within the endoplasmic reticulum of HEK293 cells. Though, WT is highly localized on the cell membrane of the HEK293 cell lines [6].

In this study, F2 carried a compound heterozygous change with a synonymous (p.Arg161Arg) and a novel frameshift (p.Val805fs) variation. The synonymous variant was reported in CHED and FECD patients [29, 30]. This variant is listed as rs3827075 in the SNP database with a global minor allele frequency of G = 0.39 (https://www.ncbi.nlm.nih.gov/snp/rs3827075). Previously, there was no evidence for CHED pathogenicity by a synonymous variant in the *SLC4A11* gene. Synonymous variations appear silent and could become pathogenic by altering codon usage, mRNA stability, or inducing translational pausing [33]. For p.Arg161Arg synonymous variation, the pathogenic or non-pathogenic effect depends on the presence of other variants. However, further studies are required to elucidate the mechanisms involved. The compound heterozygous variant severely affected one sibling and mildly affected another, showing varied expressions. The homology-based protein modelling provides a better platform to analyse the physio-chemical changes caused by the variations at various levels of structural analysis. It has been applied in this study as an alternative model to analyse mutational effects in the absence of a known crystal structure for *SLC4A11* [34]. In silico analysis showed that the identified novel p.Ser489Trp variation significantly decreased H-bond interactions around the mutational coordinate. The other identified substitution variations, S480I and R869C, showed a marginal decrease in H-bond interactions. Hydrogen bonds in a protein determine most of the directional interactions. Localised change in the number of interactions can affect protein folding, flexibility, conformation, and molecular recognition. A localised decrease in hydrogen bond interactions might affect overall protein conformation or structure. During structural analysis, the mutations compromised the proteins’ flexibility and stability. The identified variations in this study were positioned in conserved regions; therefore, they could potentially have deleterious effects. In this study, two cases were negative for *SLC4A11* variations. Few studies have shown similar results. In a study by Hemadevi B et al., 9/20 families of Indian origin did not show mutations in the coding and promoter regions of the *SLC4A11* gene [35]. All nine members of a large multi-consanguineous marriage of a CHED family from Saudi showed p.Thr271Met mutation. An affected twin family was the exception, which did not show any pathogenic mutation [36]. In another study, 9/25 CHED families from north India were characterized using *SLC4A11* mutations [37]. The remaining 11 families were negative for the *SLC4A11* mutations. These studies, including ours, emphasize the involvement of other genes or genetic mechanisms in CHED pathogenesis. Recently, exome sequencing identified a single patient with a variation in *MPDZ* (Multiple PDZ domain Crumbs cell polarity complex component) gene that might be involved in CHED pathogenesis. The patient did not have the *SLC4A11* mutation. The mother carrying the MPDZ gene variation had a mild FECD phenotype, a phenomenon seen in patients with the *SLC4A11* gene [38]. However, evidence for MPDZ in *SLC4A11* negative cases is scarce and requires further studies. The absence of *SLC4A11* mutations in CHED cases could be because of the lack of accurate differential diagnosis. The limitations of Sanger sequencing in identifying large heterozygous deletions might also affect negative results [39]. Next generation sequencing (NGS)-based analysis of *SLC4A11* negative cases from different ethnicities could provide insight into more unknown genetic factors. Further studies are needed to establish candidate genes or genetic mechanisms for CHED patients not carrying the *SLC4A11* variations.

## Conclusions

This study contributed to the CHED mutational spectrum by adding four novel variations and confirming a previously reported variation in two familial cases and three sporadic cases. Our analysis emphasizes the involvement of other genes, genetic mechanisms, or clinical diagnosis for *SLC4A11* negative CHED cases. These findings might help in planning gene editing corrections to reverse these variations and improve the management of CHED.

## Supplementary Information

**Additional file 1.** **Table S1.** List of identified variants of SLC4A11 reported in CHED/FECD phenotype. **Table S2.** List of primers and PCR annealing temperature for each amplicon of SLC4A11.

## Data Availability

All patient data has been anonymized, and any further information may be obtained from the corresponding author.

## Abbreviations

CHED: Congenital hereditary endothelial dystrophy
*SLC4A11*: Sodium bicarbonate transporter-like protein-11
FECD: Fuchs endothelial corneal dystrophy
PPCD: Posterior polymorphous corneal dystrophy
CDPD: Corneal dystrophy and perceptive deafness
AS-OCT: Anterior segment optical coherence tomography
IC3D: International classification of corneal dystrophy
NSAID: Non‐steroidal anti‐inflammatory drugs
CRISPR: Clustered regularly interspaced short palindromic repeats
BPA: Bisphenol A
