# Correction to: identification and in silico analysis of a spectrum of SLC4A11 variations in indian familial and sporadic cases of congenital hereditary endothelial dystrophy

**DOI:** 10.1186/s13023-023-02791-6

**Published:** 2023-06-29

**Authors:** Mohd Salman, Anshuman Verma, Sunita Chaurasia, Deeksha Prasad, Chitra Kannabiran, Vivek Singh, Muralidhar Ramappa

**Affiliations:** 1 Prof. Brien Holden Eye Research Center, Champalimaud Translational Centre for Eye Research, L V Prasad Eye Institute, Hyderabad, India; 2 Manipal Academy of Higher Education, Manipal, Karnataka India; 3 MNR Foundation for Research and Innovations, MNR Medical College, MNR Nagar, Sangareddy, Telangana India; 4 Centre for Rare Eye Diseases and Ocular Genetics, L V Prasad Eye Institute, Hyderabad, India; 5 Jasti V Ramanamma Children’s Eye Care Center, L V Prasad Eye Institute, Hyderabad, India; 6 The Cornea and Anterior Segment, L V Prasad Eye Institute, KAR Campus, Banjara Hills, Hyderabad, India

Following publication of the original article [1], we have been notified that the Ethics approval and consent to participate declarations should be as follows:

The study was approved by the institutional review board of the L V Prasad Eye Institute (Ethics Ref. No. LEC-BHR-01–20–381). Written informed consent from all adult participants and legal guardians/parents for minors were obtained for the research study and publications.“Ethics approval and consent to participate” section on page no. 11 to be considered as stated above. However, the statement mentioned in the Ethics statement of Methods section on page no. 2 is correct.
