# Supplementary material for: Identification and in silico analysis of a spectrum of SLC4A11 variations in Indian familial and sporadic cases of congenital hereditary endothelial dystrophy
Source: Orphanet J Rare Dis. 2022 Sep 17;17:361. doi: 10.1186/s13023-022-02521-4 (PMC9482203; doi:10.1186/s13023-022-02521-4)
Supplement: Supplementary file 1 — Additional file 1. Table S1. List of identified variants of SLC4A11 reported in CHED/FECD phenotype. Table S2. List of primers and PCR annealing temperature for each amplicon of SLC4A11. [file 13023_2022_2521_MOESM1_ESM.docx]

**Supplementary Table1: List of identified variants of *SLC4A11* reported in CHED/FECD phenotype**

| **Variants** | **Amino acid change** | **Chromosomal location** | **Exons** | **Ethnic origin** | **Clinical**  **phenotype** | | **Reference (PMID)** |
| --- | --- | --- | --- | --- | --- | --- | --- |
| c.99_100delTC | p.Ser33SerfsX18 | 20:3237580-3237584 | 1 | Chinese | | FECD | 18024964 |
| c.150T>A | p.Cys50X | 20:3234881 | 1 | Iran | | CHED2 | 31420327 |
| g.2943delTTinsA | p.Arg82ArgfsX33 | 20:3234785 -3234786 | 2 | India | | CHED2 | 16825429 |
| c.382C>T | p.Arg112X | 20:3234573 | 4 | India | | CHED2 | 31714402 |
| c.374G>A | p.Arg125His | 20:3234281 | 4 | India | | CHED2 | 18474783 |
| c.433G>A | p.Asp129Asn | 20:3234268 | 4 | India | | CHED2 | 31714402 |
| c.397T>C | p.Phe133Leu | 20:3234257 | 4 | Mexican | | CHED2 | 24351571 |
| c.427G>A | p.Glu143Lys | 20:3234227 | 4 | India | | CHED2 | 17397048 |
| c.522C>T | p.Arg158Arg | 20:3234180 | 4 | African American | | FECD | 24348007 |
| c.473_480del GCTTCGCC | p.Arg158ProfsX4 | 20:3234173-3234181 | 4 | Gipsy Eastern Europe) | | Harboyan  syndrome | 17220209 |
| Hom c.473_480 delGCTTCGCCinsC | p.Arg158ProFSX3 | 20:3234173-3234181 | 4 | India | | CHED2 | 18474783 |
| c.520delGCTTCGCC | p.Arg158fs | 20:3234173-3234181 | 4 | Saudi | | CHED2 | 19369245 |
| g.3552G>A | p.Ala160Thr | 20:3234176 | 4 | India | | CHED2 | 16825429 |
| c.478G>A | p.Ala160Trp | 20:3234176 | 4 | India | | CHED2 | 18474783 |
| c.501G>C | p.Glu167Asp | 20:3234153 | 4 | Northern Europe | | FECD | 20848555 |
| c.586G>T | p.Asp196Tyr | 20:3233986 | 4 | Iran | | CHED2 | 31420327 |
| *c.637T>C | p.Ser213Pro | 20:3233937 | 5 | Sephardi Jewish | | Harboyan  syndrome | 17220209 |
| *c.743G>A | p.Ser232Asn | 20:3233596 | 6 | Chinese-American | | CHED2 | 17667634 |
| c.720G>A | p.Trp240X | 20:3233572 | 6 | UK | | CHED2 | 17397048 |
| c.271C>T | p.Thr271Met | 20:3228299 | 6 | Saudi | | CHED2 | 18363173 |
| *c.1033A>T | p.Arg329X | 20:3231341 | 7 | Chinese-American | | CHED2 | 17667634 |
| c.806C>T | p.Ala269Val | 20:3231520 | 7 | India | | CHED2 | 18474783 |
| c.845G>C | p.Arg282Pro | 20:3231481 | 7 | Northern Europe | | FECD | 20848555 |
| c.785C>T | p.Thr262Ile | 20:3231541 | 8 | India | | CHED2 | 23922488 |
| Hom c.859_862delGAGAinsCCT | p.Glu287fsX21 | 20:3231467-3231470 | 8 | India | | CHED2 | 17262014 |
| c.1158C>A | p.Cys386* | 20:3230991 | 9 | Korean | | CHED2 | 24502824 |
| c.1156T>C | p.Cys386Arg | 20:3230993 | 9 | India | | CHED2 | 21203343 |
| *c.1156T>C | p.Cys386Arg | 20:3230991 | 9 | India | | CHED2 | 27609159 |
| c.1228G>C | p.Gly394Arg | 20:3230969 | 9 | Saudi | | CHED2 | 19369245 |
| c.1217A>T | p.Asp406Val | 20:3230934 | 9 | Iran | | CHED2 | 31420327 |
| c.1245delC | p.Ser415RfsX15 | 20:3230817 | 9 | Iran | | CHED2 | 31420327 |
| *c.1244G>A | p.Ser415Ala | 20:3230818 | 10 | India | | CHED2 | 27609159 |
| c.1253G>A | p. Gly418Asp | 20:3230809 | 10 | Saudi | | CHED2 | 19369245 |
| c.1307C>T | p.Ala436Val | 20:3230755 | 10 | Iran | | CHED2 | 31420327 |
| c.1249 G>A | p.Gly417Arg | 20:3230812 | 11 | India | | CHED2 | 23922488 |
| c.1378_1381del TACGinsA) | p.Tyr460_Ala461 delinsThr | 20:3230597-3230600 | 11 | Dominican Republic | | Harboyan  syndrome | 17220209 |
| c.1391G>A | p.Gly464Asp | 20:3230587 | 11 | Pakistan | | CHED2 | 16767101 |
| c.1391G>A | p.Gly464Asp | 20:3230587 | 11 | Pakistan | | CHED2 | 24351571 |
| c.1463G>A | p.Arg488Lys | 20:3230515 | 11 | Morocco | | Harboyan  syndrome | 17220209 |
| c.1537 + 1G>C | Splice site | 20:3230188 | 12 | Iran | | CHED2 | 31420327 |
| c.1466C>T | p.Ser489Leu | 20:3230258 | 12 | Pakistan | | CHED2 | 16767101 |
| c.1577A>G | p.Tyr526Cys | 20:3229737 | 13 | Northern Europe | | FECD | 20848555 |
| g.8118delCT | p.His568HisfsX177 | 20:3229612 -3229613 | 13 | India | | CHED2 | 16825429 |
| c.1723G>A | p.Val575Met | 20:3229591 | 13 | Northern Europe | | FECD | 20848555 |
| c.1748G>A | p.Gly583Asp | 20:3229566 | 13 | Northern Europe | | FECD | 20848555 |
| c.1813C>T | p.Arg605X | 20:3229430 | 14 | India | | CHED2 | 16767101 |
| g.8298C>T | p.Arg605X | 20:3229430 | 14 | India | | CHED2 | 16825429 |
| c.1831T>C | p.Cys611Arg | 20:3229412 | 14 | India | | CHED2 | 23922488 |
| g.8379G>T | p.Glu632X | 20:3229349 | 14 | India | | CHED2 | 16825429 |
| Hom c.2014_2016delTTC | Phe672del | 20:3229149-3229151 | 15 | India | | CHED2 | 17262014 |
| c.2114 + 1G>A | Splice site | 20:3229264 | 15 | Saudi | | CHED2 | 19369245 |
| c.2170 C>G | p.His724Asp | 20:3228908 | 16 | India | | CHED2 | 23922488 |
| c.2224G>A | p.Gly742Arg | 20:3228853 | 16 | Northern Europe | | FECD | 20848555 |
| *c.2233_2240dup TATGACAC) | p.Thr747ThrfsX6 | 20:3228838 | 16 | South American Indian | | Harboyan  syndrome | 17220209 |
| c.2236C>T | p.Arg757X | 20:3228675 | 16 | Saudi | | CHED2 | 19369245 |
| *c.2528T>C) | p.Leu843Pro | 20:3228337 | 16 | South American Indian | | Harboyan  syndrome | 17220209 |
| c.2240 + 1G>A | Splice site | 20:3228837 | 16 splice region | UK | | CHED2 | 17397048 |
| c.2240 + 1G>A | Splice site termination | 20:3228837 | 16 splice region | India | | CHED2 | 21203343 |
| c.2264G>A | p.Arg755Gln | 20:3228684 | 17 | Myanmar | | CHED2 | 16767101 |
| c.2263C>T | p.Arg755Trp | 20:3228685 | 17 | India | | CHED2 | 18474783 |
| Hom c.2318C>T | p.Pro773Leu | 20:3228630 | 17 | India | | CHED2 | 18474783 |
| *c.2398C>T | p.Gln800X | 20:3228550 | Ex17 | UK | | CHED2 | 17397048 |
| *c.2437-1G>A | Splice acceptor inactivation | 20:3228697 | Intron17 | UK | | CHED2 | 17397048 |
| *c.2566A>G | p.Met856Val | 20:3228299 | 18 | Sephardi Jewish | | Harboyan  syndrome | 17220209 |
| *c.2423_2454del32nt | p.Leu808ArgfsX  110 | 20:3228514-3228540 | 18 | Netherlands | | Harboyan  syndrome | 17220209 |
| c.2470G>A | p.Val824Met | 20:3228395 | 18 | India | | CHED2 | 31714402 |
| c.2470G>A | p.Val824Met | 20:3228395 | 18 | India | | CHED2 | 21203343 |
| c.2470G>A | p.Val824Met | 20:3228395 | 18 | India | | Non-syndromic CHED | 17220209 |
| Hom g.9361C>T | p.Thr833Met | 20:3228367 | 18 | India | | CHED2 | 16825429 |
| c.2500G>A | p.Gly834Ser | 20:3228365 | 18 | Northern Europe | | FECD | 20848555 |
| c.2518-2520delCTG | p.Leu840del | 20:3228347 - 3228349 | 18 | India | | CHED2 | 21203343 |
| *c.2528T>C | p.Leu843Pro | 20:3228337 | 18 | Netherlands | | Harboyan  syndrome | 17220209 |
| c.2605C>T | p.Arg869Cys | 20:3228260 | 18 | India | | CHED2 | 16767101 |
| Hom g.9469G>A | p.Arg869His | 20:3228259 | 18 | India | | CHED2 | 16825429 |

Variants nomenclature and chromosomal location are based on Transcript ID: ENST00000380056.7 SLC4A11-201, * represent the heterozygous variants.

**Supplementary Table2: Primer list and PCR annealing temperature for each amplicon of *SLC4A11***

| **Amplicon No.** | **Primer Sequence (5’-3’)** | | **Annealing Temp (^°^C)** | **Amplicon size (bp)** | **Reference** |
| --- | --- | --- | --- | --- | --- |
| **Amplicon 1**  Exon 1 | FP | 5’ TGAGATTAAGGCTGGCTTCC 3’ | 64 | 298 | N/A |
|  | RP | 5’CTTTTGCCCGACAAGCTCT 3’ |  |  |  |
| **Amplicon 2**  Exon 2-3 | FP | 5’ CGAGAGTGGGACAGTCCAG 3’ | 66 | 497 | N/A |
|  | RP | 5’AGGGAAGCCATCACCTCAG 3’ |  |  |  |
| **Amplicon 3**  Exon 4-5 | FP | 5’ GGCCCGTGTGGTTCTGTC 3’ | 66 | 494 | N/A |
|  | RP | 5’ACAGGGGACATGGGACAC 3’ |  |  |  |
| **Amplicon 4**  Exon 6 | FP | 5’ CAAGGTCGAGGGGGTTCT 3’ | 66 | 351 | (17) |
|  | RP | 5’ GTTTCTGACACACCCACAGG 3’ |  |  |  |
| **Amplicon 5**  Exon 7-8 | FP | 5’GGGAGAGCACCTTCACCTG 3’ | 64 | 556 | N/A |
|  | RP | 5’ GGATGGGAGAGAGGGTTTGCT 3’ |  |  |  |
| **Amplicon 6**  Exon 9-10 | FP | 5’ ACTGATGGTACGTGGCCTCT 3’ | 64 | 567 | (17) |
|  | RP | 5’ CGTCCATGCGTAGAAGGAGT 3’ |  |  |  |
| **Amplicon 7**  Exon 11-12 | FP | 5’ CATTGGTGATTCTGCTGACC 3’ | 66 | 696 | (17) |
|  | RP | 5’ ACTCAGCTTGAGCCAGTCCT 3’ |  |  |  |
| **Amplicon 8**  Exon 13-14 | FP | 5’ GAGCCCTTTCTCCCTGAGAT 3’ | 64 | 623 | (17) |
|  | RP | 5’ GGTTGTAGCGGAACTTGCTC 3’ |  |  |  |
| **Amplicon 9**  Exon 15 | FP | 5’GCCTTCTCCCTCATCAGCTC 3’ | 66 | 399 | (17) |
|  | RP | 5’ GTAGGCAGTGCCCTTCACC 3’ |  |  |  |
| **Amplicon 10**  Exon 16 | FP | 5’ AATGCACCGGAGAACAGGT 3’ | 66 | 389 | (17) |
|  | RP | 5’ CCGCGAGTGTCACCTCTG 3’ |  |  |  |
| **Amplicon 11**  Exon 17 | FP | 5’ CGTGGACCCTGAGGAGTG 3’ | 62 | 420 | (17) |
|  | RP | 5’ CCCTCCGGATGTAGTGTGTC 3’ |  |  |  |
| **Amplicon 12**  Exon 18 | FP | 5’ CTCGATGGCAACCAGCTC 3’ | 66 | 452 | (17) |
|  | RP | 5’ CTAGGCAGGACCCCTCCTC 3’ |  |  |  |
| **Amplicon 13**  Exon 19 | FP | 5’ GGTGTCCACTGCCTTCTCTC 3’ | 64 | 341 | N/A |
|  | RP | 5’ AACGCTCTTGGCCTAAAGCT 3’ |  |  |  |

**FP: Forward Primer; RP: Reverse Primer**
